# Gender‐Specific Body Areas Satisfaction and Body Weight Status in Adolescents: Mediating Effects of Physical Activity, Fruit and Vegetable Intake, and Energy‐Dense Food Intake

**DOI:** 10.1111/aphw.12145

**Published:** 2018-10-05

**Authors:** Karolina Zarychta, Carina K.Y. Chan, Magdalena Kruk, Aleksandra Luszczynska

**Affiliations:** ^1^ SWPS University of Social Sciences and Humanities in Wroclaw Poland; ^2^ La Trobe University Australia; ^3^ University of Colorado at Colorado Springs Colorado Springs USA

**Keywords:** body satisfaction, body weight, nutrition behaviour, physical activity, adolescence

## Abstract

**Background:**

Body satisfaction is one of the key modifiable cognitive determinants of eating behaviours, physical activity, and body mass index (BMI). As the sociocultural models suggest, low body satisfaction may explain unhealthy eating and exercise behaviours. Importantly, body satisfaction levels and body areas that individuals focus on vary across genders. This study aims at investigating links between the global index of body areas satisfaction (BAS), gender‐specific BAS, fruit and vegetable (F&V) intake, energy‐dense foods intake, moderate‐to‐vigorous physical activity (MVPA), and BMI.

**Methods:**

In all, 1,254 adolescents completed questionnaires and had their weight and height objectively measured with 2‐ and 13‐month follow‐ups. Indirect effects of three indices of BAS were tested in three models (male‐specific BAS amongst boys; female‐specific BAS amongst girls; the global BAS index in the total sample).

**Results:**

Higher levels of all three BAS indices indirectly predicted lower BMI, with higher MVPA mediating this effect. In addition, higher energy‐dense foods intake mediated higher global BAS–higher BMI relationship in the total sample. Thus, the global index of BAS acts as double‐edged sword, predicting both higher MVPA and energy‐dense foods intake.

**Conclusion:**

BAS may operate in a complex manner, predicting behaviours which may have opposite effects on BMI.

## Introduction

According to the Health Behaviour School‐aged Children survey (HBSC, 2017), up to 21.7 per cent of adolescents are overweight or obese (applying the International Obesity Task Force thresholds for excessive weight; Cole, Bellizzi, Flegal, & Dietz, 2000). In the last three decades, overweight and obesity have doubled in adolescents (National Center for Health Statistics, 2013). Considering European countries, Poland is the country with the sixth‐highest rates (HBSC, 2017). Excessive weight is inseparably linked to energy‐dense foods intake and physical inactivity (Spink, Wilson, & Ulvick, 2012). Therefore, the World Health Organization (WHO, 2012) guidelines on physical activity and nutrition for adolescents recommend performing at least 60 minutes of moderate‐to‐vigorous physical activity (MVPA) daily and eating at least four healthy meals a day (including fresh fruit and vegetables) in order to maintain healthy body weight. Adolescents should also eat lower energy‐dense food that provides fewer calories per gram of food as it was found to be a long‐term predictor of healthy weight (Best et al., 2016). Since overweight and obesity rates are still rising and the related health risk can be severe, the identification of modifiable risk factors for excessive weight and factors promoting healthy behaviours and healthy body weight is still of key importance in the prevention and treatment processes.

Body satisfaction is one of the key modifiable cognitive determinants of eating behaviours, physical activity, and body mass index (BMI; e.g. Perkins, Perkins, & Craig, 2010; Savage, DiNallo, & Downs, 2009). Individuals who are satisfied with their bodies are less likely to diet restrictively or use other weight control behaviours, and are more likely to be physically active and eat more fruit and vegetables (Neumark‐Sztainer, Paxton, Hannan, Haines, & Story, 2006). The transdiagnostic model of eating disorders (Fairburn, Cooper, & Shafran, 2003) suggests a specific causal chain of effects, starting with body dissatisfaction that leads to changes in energy expenditure behaviours (e.g. dieting or excessive physical activity) that in turn determine unfavourable shifts in body mass. Body concerns are present as early as in 5‐year‐olds, and from that time on body satisfaction is assumed to decrease, being the lowest during adolescence (Bratovcic, Mikic, Kostovski, Teskeredzic, & Tanovic, 2015). Therefore, body satisfaction is especially important among adolescents and should be included in studies testing predictors of BMI and heathy behaviours.

The sociocultural models (Striegel‐Moore & Bulik, 2007) suggest that cultural pressure to achieve an unattainable ideal figure leads to dissatisfaction with one's body. In turn, lower body satisfaction has been found to be a predictor of disordered eating or improper weight control behaviours (Westerberg‐Jacobson, Ghaderi, & Edlund, 2012). Moreover, the sociocultural model highlights the gender differences in ideal body and body areas satisfaction (BAS). These differences are assumed to be consequences of society and media emphasising higher importance of body weight, body shape, and appearance in general in women than in men (Cash, Morrow, Hrabosky, & Perry, 2004), with physical attractiveness being linked to professional success and competence, happiness, youth, and good health. Lower body satisfaction is so common in women that it is perceived to be a normative component of their lives (Kostanski, Fisher, & Gullone, 2004). Thus, it was indicated that women are less satisfied with their bodies (Cash et al., 2004) and in consequence eat less fruit and vegetables (F&V) or more energy‐dense foods, and are less physically active than men (Paxton, Neumark‐Sztainer, Hannan, & Eisenberg, 2006). Research has confirmed that body satisfaction is important also in men, though (Kostanski et al., 2004). Gender differences can be found in ideal body weight and shape with women desiring lean and low in body fat physiques, and men wanting lean and high in muscles physiques (Heinberg et al., 2008). Studies indicated that women want to lose weight regardless of their BMI, but men are divided into fairly equal groups of those who are overweight or obese and want to lose weight, and those who are underweight and want to gain weight (Kostanski et al., 2004). Moreover, body areas that individuals are focused on vary across genders, with women being preoccupied with the mid and lower torso (hourglass figure), and men with the upper and mid torso (V‐shaped figure). Satisfaction with specific body areas is associated with general or overall body satisfaction in both genders (Mellor et al., 2014).

Due to the above‐mentioned gender differences, there is a need to test body satisfaction and its effects in men and women separately (Mäkinen, Puukko‐Viertomies, Lindberg, Siimes, & Aalberg, 2012). However, if the complex effects of BAS are investigated in separate samples and using distinct BAS indicators, it is not possible to strictly test if gender significantly differentiates these associations. This can be obtained when both genders are included in one model and gender is assumed to act as a moderator. Therefore, in the present study, two gender‐specific types of body areas satisfaction (BAS) were used to test the associations between variables in two separate samples of boys and girls. In addition, it has been tested whether the effects of general BAS were different for boys and girls using gender as a covariant and a moderator in respective analyses.

Results of multiple cross‐sectional and rare longitudinal studies confirmed associations between body satisfaction, healthy nutrition behaviour indicators such as F&V intake (e.g. Balluck, Toorabally, & Hosenally, 2016), and physical activity (e.g. Laus, Braga Costa, & Almeida, 2011). Importantly, most of this research tested only direct associations between BAS, nutrition behaviour, and physical activity indicators, and BMI (e.g. Coelho, Giatt, Molin, Nunes, & Barreto, 2015). Indirect effects of energy expenditure behaviours mediating the relationship between BAS and body mass were not investigated. Moreover, to the best of our knowledge, there are no studies accounting for the association between body satisfaction and energy‐dense foods intake predicting adolescents’ BMI. Reducing energy‐dense food is a main factor responsible for unfavourable shifts in body mass (Best et al., 2016). Furthermore, previous studies were most often conducted in small samples of adolescents (Balluck et al., 2016; Laus et al., 2011) and used self‐reported measures of body mass (Penkal & Kurdek, 2007).

To fully understand temporal effects amongst body satisfaction, F&V or energy‐dense food intake, MVPA, and BMI, variables should be measured at separate time points (MacKinnon, 2008). Unfortunately, as mentioned above, most research so far has had a cross‐sectional design. Moreover, the majority of previous research referred to clinical samples or overweight and obese samples of adolescents (Sonneville et al., 2012), suggesting that promoting body satisfaction might be the most beneficial for weight loss or treating disordered eating. Indeed, several psychosocial factors (such as body dissatisfaction or stigmatisation) may play a particularly salient role in explaining maintenance of disorder symptoms or unhealthy eating patterns amongst adolescents with eating disorders or those with excessive weight (Striegel‐Moore & Bulik, 2007). Still, it can be assumed that an investigation of the associations amongst body satisfaction, physical activity, F&V and energy‐dense food intake, and BMI should account for adolescents in general (Neumark‐Sztainer et al., 2006). The present study fills that gap by investigating body satisfaction in adolescents with a wide range of BMI. Moreover, three measurement points were used to establish temporal precedence, and direct and indirect effects of body satisfaction on behaviours and objectively measured BMI were investigated.

The aim of this prospective study was to investigate the associations between three indices of body areas satisfaction (male‐specific BAS, female‐specific BAS, and a global index of BAS), F&V intake, energy‐dense food intake (i.e. fatty foods and sweets), MVPA, and BMIs of adolescents from the general population. The indirect effects of male‐specific BAS were tested amongst boys, and indirect effects of female‐specific BAS were tested amongst girls. We hypothesised that all three indices of BAS may be indirectly related to lower adolescent BMI through F&V intake and MVPA, and to higher adolescent BMI through energy‐dense food intake. In particular, we hypothesised that:

1. the association between male‐specific BAS (Time 1; T1) and BMI *z*‐scores (T3) would be mediated by MVPA (Time 2; T2), F&V intake (T2), and energy‐dense food intake (T2) in boys (Hypothesis 1; H1);
2. the association between female‐specific BAS (T1) and BMI *z*‐scores (T3) would be mediated by MVPA (Time 2; T2), F&V intake (T2), and energy‐dense food intake (T2) in girls (H2);
3. the association between global index of BAS (T1) and BMI *z*‐scores (T3) would be mediated by MVPA (Time 2; T2), F&V intake (T2), and energy‐dense food intake (T2) in adolescents (H3); it was also explored whether this indirect effect would be moderated by participants’ gender.

All hypotheses were tested controlling for the effects of age and gender (when appropriate) on the dependent variable.

## Methods

### Participants

At Time 1 (T1; baseline), 1,254 adolescents (58.3% girls) aged 13–18 years old (*M *=* *16.37, *SD *=* *.78) with BMIs ranging from 15.59 to 40.35 (*M *=* *21.99, *SD *=* *3.26) participated in the study, of whom eight (0.6%) adolescents were underweight, 988 (78.8%) had normal body weight, 208 (16.6%) were overweight, and 50 (4.0%) obese. At Time 2 (T2; 2‐month follow‐up), a total of 1,093 (55.1% girls) adolescents aged 13–19 years old (*M *=* *16.58, *SD *=* *.81) with BMIs ranging from 15.61 to 38.54 (*M *=* *21.78, *SD *=* *3.17) provided their data. At T2, eight (0.7%) participants were underweight, 886 (81.1%) had normal body weight, 158 (14.5%) were overweight, and 41 (3.7%) obese. At Time 3 (T3; 13‐month follow‐up), 986 (53.6% girls) adolescents aged 14–19 years old (*M *=* *17.45, *SD *= .80) with BMIs ranging from 14.54 to 38.85 (*M *=* *21.17, *SD *= 3.09) took part in the study, with six (0.6%) adolescents being underweight, 845 (85.7%) normal body weight, 115 (11.7%) overweight, and 20 (2.0%) obese.

All participants were Caucasian. The majority (64%) lived in urban areas, with 36 per cent living in rural areas. Data were collected in three regions of Poland representing three levels of the mean household income: the national average (Southern Region), the highest national level (Central Region), and the lowest national level (Eastern Region) (Central Statistics Office, 2017).

The total attrition rate was 21.37 per cent. Any missing data were imputed, including data missing due to the longitudinal dropout and data missing for specific items at any measurement points. Therefore, data collected from *N *=* *1,254 adolescents (58.3% girls) aged 13 to 18 years old (*M *=* *16.37, *SD* = .78) with BMIs ranging from 15.59 to 40.35 (*M *=* *21.99, *SD* = 3.26) were included in the analyses.

### Procedure

The study was conducted in 16 public middle and high schools. All respondents lived with their parents (98.9%) or other legal guardians (1.1%) at all measurement points. Participants and parents of those younger than 18 years old provided informed consent prior to the data collection. Individuals were informed about the objectives and the procedure of the study, and were assigned personal codes to secure anonymity and identification across the measurement points. Participants provided their data referring to their nutrition behaviours, MVPA, and body areas satisfaction. At T1, each participant filled in a questionnaire. Afterwards, their height and weight were measured privately by researchers in another room or in the school nurse's office. This procedure was repeated at T2 and T3. Researchers were available for consultations after study completion, if desired. Multiple efforts were made to reduce attrition, and at T2 and T3, researchers returned 3–5 times across a three‐week period in order to gain access to participants who were willing to respond but were temporarily absent. Attrition rate was associated primarily with finishing, changing, or dropping out of school by participants. The study was approved by the Institutional Review Board at the first author's university. All procedures were in accordance with the ethical standards of the institutional research ethics committee and in line with the 1964 Helsinki Declaration and its later amendments.

### Materials

Means, standard deviations, results of variance analyses, and reliability coefficients are presented in Table 1. Validity of the measures used in the study has been confirmed elsewhere (i.e. Brytek‐Matera & Rogoza, 2015; Murphy, Rowe, Belton, & Woods, 2015; Yaroch et al., 2012).

**Table 1:** Descriptive Statistics, Between‐Groups Differences, and Reliability of the Study Variables at T1, T2, and T3 (*N *=* *1,254)

		Differences between boys and girls: F	M (SD) for boys / M (SD) for girls	Total sample: M (SD)	α
1	T1 Male‐specific body areas satisfaction	2.44	3.16 (.73) / 3.45 (.68)	3.28 (.72)	.79
2	T1 Female‐specific body areas satisfaction	2.41	3.45 (.72) / 3.19 (.78)	3.30 (.77)	.79
3	T1 Global index of body areas satisfaction	3.09	3.45 (.74) / 3.14 (.68)	3.27 (.73)	.82
4	T2 Moderate‐to‐vigorous physical activity	21.16***	6.69 (3.68) / 5.67 (3.03)	6.10 (3.35)	.60
5	T2 Fruit and vegetable intake	3.86*	5.58 (2.06) / 5.88 (2.23)	5.76 (2.17)	.74
6	T2 Energy‐dense food intake	6.00*	4.42 (1.91) / 3.77 (1.73)	4.04 (1.84)	.71
7	T1 Body mass index *z*‐scores	6.08**	.38 (.87) / .19 (1.00)	.27 (.95)	
8	T3 Body mass index *z*‐scores	16.48***	.10 (.88) / − .41 (1.09)	− .20 (1.04)	
9	T1 Age	35.46	16.37 (.66) / 16.36 (.92)	16.37 (.78)	
10	Gender				

*Note*

T1 = Time 1, baseline; T2 = Time 2, 2‐month follow‐up; T3 = Time 3, 13‐month follow‐up.

***p <* *.001; **p <* *.01; *p <* *.05.

#### Three Indices of Body Areas Satisfaction (Male‐Specific BAS, Female‐Specific BAS, and Global Index of BAS) (T1)

All three indices of body areas satisfaction were measured based on the Multidimensional Body‐Self Relations Questionnaire's Body Areas Satisfaction Subscale (MBSRQ; Cash, 2000). The measure assesses feelings of physical attractiveness or unattractiveness with discrete aspects of one's appearance. In order to assess a global index of BAS, the respondents were asked to indicate how dissatisfied or satisfied they were with the nine following areas of their body: face (facial features, complexion), hair (colour, thickness, texture), lower torso (buttocks, hips, thighs, legs), mid torso (waist, stomach), upper torso (chest or breast, shoulders, arms), muscle tone, weight, height, and overall appearance. The responses ranged from 1 (*very dissatisfied*) to 5 (*very satisfied*). High scores relate to individuals generally content with most areas of their body and low scores relate to individuals unhappy with several areas of their body or its size.

In addition, the MBSRQ's Body Areas Satisfaction Subscale was divided into two subscales regarding gender‐specific BAS: (1) male‐specific BAS (consisting of mid torso, upper torso, muscle tone, weight, height, face, hair, and overall appearance), and (2) female‐specific BAS (consisting of mid torso, lower torso, weight, face, hair, and overall appearance). The selection of male‐ and female‐specific BAS was made based on previous research (e.g. Frederick et al., 2007; Mellor et al., 2014; Penkal & Kurdek, 2007).

#### Fruit and Vegetable Intake (T2)

In order to evaluate F&V intake, adolescents answered two questions, adapted from Lally, Bartle, and Wardle (2011): “How often did you eat a portion of fresh fruit in the last two weeks?” and “How often did you eat a portion of vegetables in the last two weeks (fresh, boiled or fried without fat)?” The portion was defined as the amount fitting into a cupped hand. The responses were given on a 6‐point scale, ranging from 1 (*once a week or less*) to 6 (*four or more times a day*). Both items were summed to give the total score for F&V intake.

#### Energy‐Dense Food Intake (T2)

In order to evaluate the intake of energy‐dense foods, adolescents were asked two questions, adapted from Lally et al. (2011): “How often did you eat fatty foods (e.g. pizza, chips, foods with dressings) in the last two weeks?” and “How often did you eat sweets (e.g. chocolate bars or wafers, cakes) in the last two weeks?” The responses were given on a 6‐point scale, ranging from 1 (*once a week or less*) to 6 (*four or more times a day*). Both items were summed to give the total score for energy‐dense food intake.

#### Moderate‐to‐Vigorous Physical Activity (T2)

MVPA was measured with two items, based on the Godin Leisure‐Time Exercise Questionnaire (Godin & Shephard, 1997). To assess MVPA levels of respondents, they were asked to consider the previous two weeks and provide the daily number of “intensive (heart beats faster, you are sweating) PA sessions lasting at least 15 minutes” and “moderate (not so exhausting) PA sessions lasting at least 15 minutes”. Examples of moderate and intensive PA were provided. Responses were open ended. Both answers were combined since MVPA is recommended for children and adolescents to achieve maximum health benefits (cf. National Institute for Health and Clinical Excellence, 2009).

#### Body Weight and Height (T1 and T3)

Biometric measures were assessed with standard medically approved telescopic height measuring rods and floor scales (scale type: BF‐100 or BF‐25). Age‐ and gender‐specific BMI percentiles were calculated with the WHO AnthroPlus macro (WHO, 2011), which is a software package for the global application of the WHO growth reference (de Onis et al., 2007) for children and adolescents. Thus, the BMI indicator of each participant accounts for their age and gender. BMI *z*‐scores were calculated and used as dependent variables in all analyses.

### Data Analysis

Data were analysed using SPSS version 24 and PROCESS macro (Model 4) with 10,000 bootstraps (Hayes, 2013). Three separate multiple mediation analyses were performed to test the associations between body areas satisfaction (male‐specific BAS, female‐specific BAS, and global index of BAS), F&V intake, energy‐dense food intake, MVPA, and adolescents’ BMIs accounting for the covariates (T1 BMI and age or T1 BMI, age, and gender). Two types of coefficients present the results of the analyses: (1) a regression coefficient for each parameter, and (2) the indirect effect coefficient (*B*) for each indirect pathway between the independent variable (IV) (T1 body areas satisfaction [male‐specific BAS, female‐specific BAS, and global index of BAS]) and the dependent variable (DV) (T3 BMI), accounting for respective mediators and covariates. Moreover, moderated multiple mediation analyses were conducted for the total sample and the global BAS index, using PROCESS macro (Model 59) to test whether the associations between (1) the IVs and the DV, (2) the IVs and the mediators, and (3) the mediators and the DV are moderated by gender.

**Figure 1:**
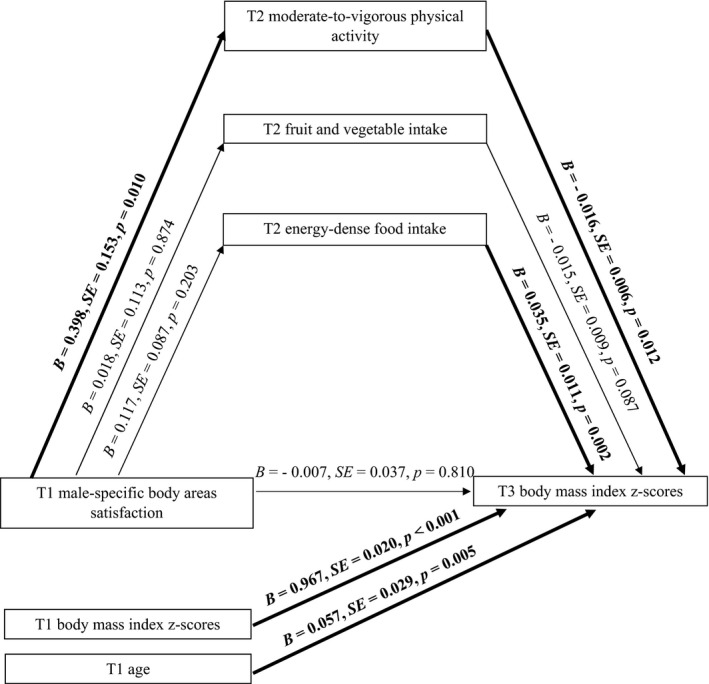
Effects of male‐specific body areas satisfaction on boys’ BMI through moderate‐to‐vigorous physical activity, fruit and vegetable intake, and energy‐dense food intake. *Note*: T1 = Time 1, baseline; T2 = Time 2, 2‐month follow‐up; T3 = Time 3, 13‐month follow‐up. Paths marked in bold represent significant associations.

**Figure 2:**
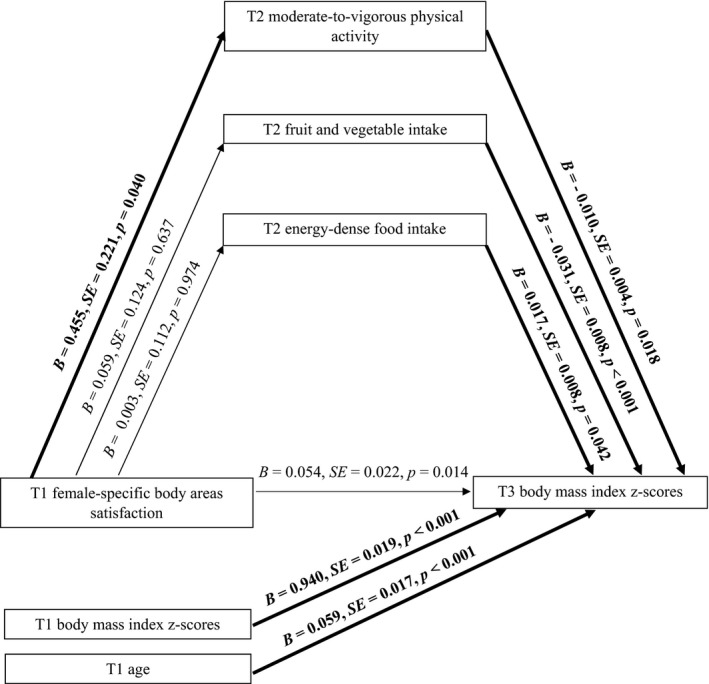
Effects of female‐specific body areas satisfaction on girls’ BMI through moderate‐to‐vigorous physical activity, fruit and vegetable intake, and energy‐dense food intake. *Note*: T1 = Time 1, baseline; T2 = Time 2, 2‐month follow‐up; T3 = Time 3, 13‐month follow‐up. Paths marked in bold represent significant associations.

**Figure 3:**
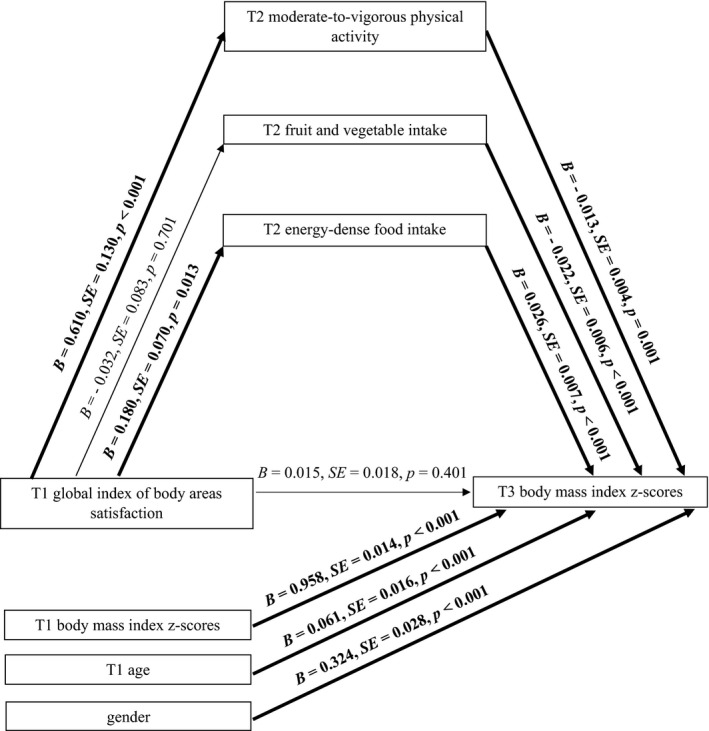
Effects of global index of body areas satisfaction on adolescents’ BMI through moderate‐to‐vigorous physical activity, fruit and vegetable intake, and energy‐dense food intake. *Note*: T1 = Time 1, baseline; T2 = Time 2, 2‐month follow‐up; T3 = Time 3, 13‐month follow‐up. Paths marked in bold represent significant associations.

In this study the IV was the global index of BAS (T1) or male‐specific BAS (T1) or female‐specific BAS (T1); the DV was the BMI *z*‐score measured at T3; the mediators were F&V intake (T2), energy‐dense food intake (T2) and MVPA (T2); the moderator was gender (T1) coded as −1 for boys and +1 for girls. The IV, the mediators, and the DV in the respective analyses were measured at different time points (T1, T2 and T3) to establish temporal precedence (MacKinnon, 2008). Missing data were imputed with the multiple imputation method. A total of 4.8 per cent of the completers’ data were missing. The attrition analysis is presented below.

## Results

### Preliminary Analyses

#### Attrition Analysis

Completers did not differ from those who dropped out at T2 or T3 in terms of all three indices of the body areas satisfaction, F&V intake, energy‐dense food intake, MVPA, and BMI, all *F*s < 6.02, *p*s > .17, or gender, *χ* ^2^ (1) = .22, *p *=* *.88. Dropouts and completers differed in terms of age, *F*(1, 1,254) = 4.96, *p *<* *.0001, with dropouts being slightly older (*M *=* *17.65, *SD* = .60) than completers (*M *=* *17.13, *SD* = .95, Cohen's *d *=* *.68, 95% CI [0.63 to 0.72]).

#### Correlation Analyses and Comparisons between Boys and Girls

Correlations between study variables for the study sample (*N *=* *1,254) are presented in Table 2. There were strong positive correlations between the global index of BAS (T1) with both male‐ and female‐specific BAS (T1). All three indices of BAS (T1) were weakly and positively associated with MVPA (T2), energy‐dense food intake (T2) and gender, and weakly and negatively related to BMI *z*‐scores (T1 and T3). There were also weak positive correlations between MVPA (T2) with F&V intake (T2) and gender. F&V intake (T2) was weakly and negatively associated with energy‐dense food intake (T2), and weakly and positively related to gender. On the other hand, energy‐dense food intake (T2) was weakly and negatively correlated with BMI *z*‐scores (T1 and T3), and weakly and positively linked to gender (see Table 2).

**Table 2:** Correlations between the Study Variables at T1, T2, and T3 (*N *=* *1,254)

		2	3	4	5	6	7	8	9	10
1	T1 Male‐specific body areas satisfaction	.73***	.78***	.13***	.01	.06*	−.14***	−.07**	.04	.19***
2	T1 Female‐specific body areas satisfaction		.76***	.10***	−.01	.06*	−.14***	−.09**	.04	.16***
3	T1 Global index of body areas satisfaction			.13***	−.01	.07*	−.14***	−.08**	.04	.21***
4	T2 Moderate‐to‐vigorous physical activity				.14***	−.04	.04	.01	−.01	.15***
5	T2 Fruit and vegetable intake					−.11***	−.09**	.02	.05†	.07*
6	T2 Energy‐dense food intake						−.17***	−.07**	−.03	.17***
7	T1 Body mass index *z*‐scores							.88***	−.04	−.10**
8	T3 Body mass index *z*‐scores								.01	.25***
9	T1 Age									−.01
10	Gender									

*Note*

T1 = Time 1, baseline; T2 = Time 2, 2‐month follow‐up; T3 = Time 3, 13‐month follow‐up.

***p <* *.001; **p <* *.01; *p <* *.05.

Next, gender differences in study variables were tested. Significant differences were found between boys and girls in terms of MVPA (T2), F&V intake (T2), energy‐dense food intake (T2), and BMI *z*‐scores (T1 and T3), with boys being more physically active and eating more energy‐dense food, and girls eating more F&V, all *F*s < 3.86, *p*s < .05 (see Table 1). No differences were found regarding all three indices of BAS (T1), all *F*s < 2.41, *p*s > .32. Moreover, participants recruited in the three regions of Poland representing three levels of household income did not differ in any of the study variables.

### Indirect Effect of Male‐Specific BAS on BMI: MVPA and Nutrition Behaviour as Mediators in Boys

H1 tested the indirect effect of *male‐specific BAS* (T1) (IV) on *BMI z‐scores* (T3) (DV) via *MVPA* (T2), *F&V intake* (T2), and *energy‐dense food intake* (T2) (see Figure 1). Analysis was performed for the boys sample (*n* =* *524). H1 was tested controlling for boys’ age and BMI *z*‐scores at T1.

The results indicated a significant indirect effect of *male‐specific BAS* (T1) on *boys’ BMI z‐scores* (T3) through *MVPA* at T2 (see Model 1 in Table 3). We also found direct relations between IV (T1), *MVPA* (T2) and DV (T3). Boys who were generally content with most areas of their body (T1) were also more physically active (T2), which predicted lower BMI *z*‐scores at T3. There were no mediating effects of *F&V intake* (T2) or *energy‐dense food intake* (T2), nor direct associations between IV, mediators, and DV.

**Table 3:** Effects of Three Indices of Body Areas Satisfaction on Adolescents’ BMI through Moderate‐to‐Vigorous Physical Activity, Fruit and Vegetable Intake, and Energy‐Dense Food Intake

*Indirect effects pathways*		B	SE	BC 95% CI	
				Lower	Higher
Testing the effect of male‐specific body areas satisfaction amongst boys (H1).					
Model 1	**T1 Male‐specific body areas satisfaction ➔ T2 moderate‐to‐vigorous physical activity ➔ T3 Body mass index ** ***z*** **‐scores**	**−.006**	**.004**	**−.016**	**−.001**
	T1 Male‐specific body areas satisfaction ➔ T2 fruit and vegetable intake ➔ T3 Body mass index *z*‐scores	−.003	.002	−.006	.003
	T1 Male‐specific body areas satisfaction ➔ T2 energy‐dense food intake ➔ T3 Body mass index *z*‐scores	.004	.004	−.002	.014
Testing the effect of female‐specific body areas satisfaction amongst girls (H2).					
Model 2	**T1 Female‐specific body areas satisfaction ➔ T2 moderate‐to‐vigorous physical activity ➔ T3 Body mass index ** ***z*** **‐scores**	**−.005**	**.003**	**−.013**	**−.001**
	T1 Female‐specific body areas satisfaction ➔ T2 fruit and vegetable intake ➔ T3 Body mass index *z*‐scores	−.002	.004	−.010	.006
	T1 Female‐specific body areas satisfaction ➔ T2 energy‐dense food intake ➔ T3 Body mass index *z*‐scores	−.001	.002	−.004	.005
Testing the effect of global index of body areas satisfaction amongst the general sample (H3).					
Model 3	**T1 Global index of BAS ➔ T2 moderate‐to‐vigorous physical activity ➔ T3 Body mass index ** ***z*** **‐scores**	**−.010**	**.003**	**−.014**	**−.003**
	T1 Global index of BAS ➔ T2 fruit and vegetable intake ➔ T3 Body mass index *z*‐scores	.001	.002	−.003	.005
	**T1 Global index of BAS ➔ T2 energy‐dense food intake ➔ T3 Body mass index ** ***z*** **‐scores**	**.005**	**.003**	**.001**	**.011**

*Note*

Values of indirect effect coefficient (*B*) presented in bold are significant. Each bootstrap was based on 10,000 repetitions. Bias corrected (BC) confidence intervals (CI) that do not include zero indicate a significant indirect effect.

T1 = Time 1, baseline; T2 = Time 2, 2‐month follow‐up; T3 = Time 3, 13‐month follow‐up; H = Hypothesis.

Significant coefficients are marked in bold.

### Indirect Effect of Female‐Specific BAS on BMI: MVPA and Nutrition Behaviour as Mediators in Girls

H2 tested the indirect effect of *female‐specific BAS* (T1) (IV) on *BMI z‐scores* (T3) (DV) via *MVPA* (T2), *F&V intake* (T2), and *energy‐dense food intake* (T2) (see Figure 2). Analysis was performed for the subsample of girls (*n *=* *732). H2 was tested controlling for girls’ age and BMI *z*‐scores (T1).

The results showed that the association between *female‐specific BAS* (T1) and *girls’ BMI z‐scores* (T3) was mediated by *MVPA* (T2) as indicated by the significant indirect effect (see Model 2 in Table 3). Direct relations between IV (T1), *MVPA* (T2), and DV (T3) were also found. Girls who were generally content with most areas of their body (T1) were also more physically active (T2), which predicted lower BMI *z*‐scores at T3. No mediating effects of F&V intake (T2) or energy‐dense food intake (T2), nor direct associations between IV, tested behaviours, and DV were found.

### Indirect Effect of the Global Index of BAS on BMI: MVPA and Nutrition Behaviour as Mediators in Adolescents

H3 tested the indirect effect of *global index of BAS* (T1) (IV) on *adolescents’ BMI z‐scores* (T3) (DV) via *MVPA* (T2), *F&V intake* (T2), and *energy‐dense food intake* (T2) (see Figure 3). Analysis was performed for the total sample (*N *=* *1,256), controlling for adolescents’ age, gender, and BMI *z*‐scores (T1).

The results showed that the association between *global index of BAS* (T1) and *adolescents’ BMI z‐scores* (T2) was mediated by *MVPA* (T2) and *energy‐dense food intake* (T2) as indicated by the significant indirect effects (see Model 3 in Table 3). Adolescents who were generally content with most areas of their body (T1) were more physically active (T2), which in turn predicted lower BMI *z*‐scores at T3. A different pattern of findings was observed for energy‐dense food intake: there was a significant indirect effect of the global index of BAS (T1) on BMI *z*‐scores at T3, but in this case a higher global index of BAS (T1) predicted eating energy‐dense food more frequently (T2), which in turn predicted higher BMI *z*‐scores at T3.

No mediating or direct effects of F&V intake (T2) were found. Direct associations between IV (T1), *MVPA* (T2), *energy‐dense food intake* (T2), and DV (T3) were also found, indicating that a higher global index of BAS (T1) was a predictor of both MVPA (T2) and energy‐dense food intake (T2), which in turn predicted lower or higher BMI (T3), respectively.

Additional moderated multiple mediation analysis, assuming that the associations between global index of BAS, MVPA, F&V and energy‐dense food intake, and adolescents’ BMI *z*‐scores were moderated by gender, indicated that these associations were the same for girls and boys.

## Discussion

This study provides novel evidence for the prospective associations between three indices of body areas satisfaction, F&V and energy‐dense food intake, moderate‐to‐vigorous physical activity, and objectively measured BMI in adolescents from the general population. For the gender‐specific BAS, the results show that both male‐ and female‐specific BAS are indirectly associated with boys’ and girls’ BMI through MVPA, but not through F&V or energy‐dense food intake. In particular, boys who were satisfied with male‐specific body areas (T1) and girls who were satisfied with female‐specific body areas (T1) were more physically active at T2, which in turn predicted their lower BMIs at T3. Regarding the global index of BAS, the results suggest that it is indirectly linked to adolescents’ BMI via MVPA and energy‐dense food intake, but not via F&V intake. These two behavioural mediators have distinct effects: adolescents satisfied with their bodies at T1 were more likely to expend energy through MVPA at T2 which in turn predicted lower BMI at T3, but at the same time they were more likely to eat more energy‐dense foods a T2 and have higher BMI at T3. No moderating effect of gender was found.

The first two hypotheses suggested that gender‐specific BAS may have indirect effects (through energy intake and expenditure behaviours) on adolescents' BMI *z*‐scores. These hypotheses have been partially confirmed. Gender‐specific BAS are useful in predicting boys’ and girls’ MVPA and BMI *z*‐scores, but not F&V or energy‐dense food intake. Importantly, the same behaviour operated as the mediator between gender‐specific BAS and BMI *z*‐scores for both boys and girls. These findings are partially in line with some of the previous studies which did not support gender differences in body satisfaction models (e.g. Penkal & Kurdek, 2007) and highlighted the need to include gender as a moderator or covariant rather than creating gender‐specific models. Our study contributes to this discussion showing that there may be a need for gender‐specific models of BAS, yet these models may differ in the content of BAS only, whereas the ways through which these gender‐specific BAS indicators operate may be similar and gender‐independent.

The third hypothesis tested the indirect effect of the global index of BAS on BMI *z*‐scores through MVPA, F&V and energy‐dense food intake in adolescents controlling for the effect of gender. Based on previous research, it could be expected that the observed effects would be different for boys and girls, with stronger associations between BAS, nutrition behaviours, and MVPA amongst girls compared with boys (e.g. Cash et al., 2004; Paxton et al., 2006). The results of the present study did not support this hypothesis. As expected, preliminary analyses indicated that girls were significantly less physically active, but ate F&V more frequently than boys, and boys ate energy‐dense food more frequently than girls. However, no significant gender differences were found in BAS. No effects of gender were found in the moderated mediation model, though. Gender was not a moderator in the associations between BAS, MVPA, F&V and energy‐dense food intake, and adolescents’ BMI *z*‐scores, which means that the associations between study variables were the same for both genders. Thus, the conclusion from our study is that when using a global index of BAS (containing both gender‐specific and unspecific BAS items), the patterns of associations may be the same for both genders. These findings are partially in line with Penkal and Kurdek (2007), as we found that gender is a significant covariate yet not a moderator of the associations.

For the total sample, we found an indirect effect of global index of BAS on BMI *z*‐scores through MVPA and energy‐dense food intake, but not through F&V intake. It is, however, important to highlight that these two mediators operate in different manners, with MVPA predicting lower BMI and energy‐dense food intake predicting higher BMI. Previous studies investigated the effects of healthy nutrition behaviour only, and indicated that adolescents satisfied with their bodies eat more F&V (e.g. Balluck et al., 2016). The effect obtained for energy‐dense food intake and MVPA can be explained by the compensatory health beliefs model (Rabia, Knäuper, & Miquelon, 2006) assuming that people believe that the negative consequences of unhealthy behaviours can be compensated for by engaging in healthy behaviours (Radtke, Scholz, Keller, & Hornung, 2012). Thus, adolescents may think that being physically active makes it possible to eat more energy‐dense foods, even though previous research (Vispute, Smith, LeCheminant, & Hurley, 2011) showed that people cannot out‐exercise unhealthy nutrition behaviour. Moreover, BAS can facilitate compensatory health behaviours as individuals’ satisfaction may operate as motivation to engage in a compensatory act (e.g. “Since I am satisfied with myself and physically active, I may reward myself with a small snack”) (Rabia et al., 2006). Summing up, the global index of BAS may act as a double‐edged sword predicting energy expenditure through MVPA and energy‐dense food intake at the same time.

In contrast to previous cross‐sectional research and studies that explored direct associations between variables (e.g. Balluck et al., 2016; Coelho et al., 2015), our investigation allowed for a more thorough prospective testing of associations between BAS, F&V and energy‐dense food intake, MVPA, and objectively measured BMI *z*‐scores in adolescents from the general population. Thus, our research expands on previous research conducted in clinical populations of adolescents or only in groups of underweight or overweight and obese adolescents (Sonneville et al., 2012), and shows that these associations seem to be consistent across the range of body weight status and in a large sample of both boys and girls.

There were, however, some limitations to the study, with the major one being the fact that we did not account for other factors previously found to mediate between BAS and BMI, such as perfectionism, negative affect, mental disorders (e.g. depression, anxiety disorders), self‐esteem, emotion regulation, or socioeconomic status (Alvarez‐Galvez & Gomez‐Baya, 2017; Sim & Zeman, 2006). Future research may test whether the findings of the present study would hold when controlling for these factors. Moreover, the associations between adolescents’ BAS, food intake, and physical activity may be explained by the shared environment (e.g. built physical environment, food accessibility) (Luszczynska et al., 2013) which should be controlled for in future research. The results may be affected by the specificity of the target group, that is, adolescents from the general population. The aim of this study, however, was to provide insight into potential mechanisms of MVPA, F&V and energy‐dense food intake in the associations between BAS and BMI *z*‐scores. Thus, a clinical population would not have been appropriate to answer this research question. Any conclusions may refer to a general population, which probably includes adolescents with a wide range of BMI and symptoms of different disorders including eating disorders. Therefore, it is worth highlighting that in clinical samples (e.g. with depression or eating disorders) the benefits of being satisfied with one's body may outweigh its drawbacks (Presnell, Bearman, & Madelley, 2007). Yet, our results for adolescents from the general population are ambiguous, indicating the relevance of both benefits and drawbacks of body satisfaction, in particular in the context of the maintenance of healthy body mass. Another limitation may be the broad age range of the sample which can limit the ability to indicate differences across younger and older adolescents. However, age was controlled for in all analyses to overcome this issue and no significant effects were found. Also, the sample analysed was ethnically homogeneous (all participants were Caucasian). Therefore, generalisations to ethnically diverse populations should be made with caution. Further, beta coefficients obtained in our study suggested weak associations between the variables. One possible explanation for this is the fact that we controlled for variables that were found to be significant confounds of adolescents’ BMI. Also, it is worth noting that the association between variables used in our study can have a different order of prediction, that start from unhealthy body mass which explains MVPA, F&V and energy‐dense food intake, that in turn predict body satisfaction (e.g. Balluck et al., 2016). Unfortunately, we have no data to test the order of the directionality of the associations. In line with previous research (e.g. Luszczynska et al., 2013), the measures used to assess MVPA, F&V and energy‐dense food intake were short. The content of the respective constructs might be better covered if the measures were more extensive; however, longer measures may lead to higher attrition and these competing demands need to be considered. Still, it should be noted that the reliability of these measures was acceptable. Moreover, to secure the internal validity and lower the chance of confounding, the study controlled for baseline BMI as well as participants’ basic sociodemographic characteristics (age and gender). Finally, it is possible that social desirability has contributed to potential biases in the participants’ responses.

In conclusion, the results of this study confirmed associations between BAS, MVPA, energy‐dense food intake, and adolescents’ BMI *z*‐scores. The findings may suggest that the inclusion of cognitive factors such as BAS in screening and obesity prevention programmes is justified and should continue. Our findings suggest that such programmes might target adolescents with a wide range of BMI *z*‐scores and of both genders. Finally, a global index of BAS may be an indicator for both stimulating energy expenditure by MVPA and energy intake (in the form of energy‐dense foods). The discovery of this ambiguous effect suggests that future research needs to carefully test how other energy‐expenditure related behaviours (e.g. sedentary behaviours) may be stimulated by BAS.

## Funding

The preparation of this paper was supported by grant 2017/01/X/HS6/00620 awarded by the National Science Centre, Poland to Karolina Zarychta. The contribution of Aleksandra Luszczynska was supported with NN 106 012240 grant from the National Science Centre, Poland.

## Conflict of Interest

The authors declare that they have no conflict of interest.
